# What clinicians need to know about intranasal esketamine for treatment-resistant depression?

**DOI:** 10.1177/10398562231211171

**Published:** 2023-11-14

**Authors:** Judy Hope, David Copolov, John Tiller, Megan Galbally, Malcolm Hopwood, Richard Newton, Nicholas A Keks

**Affiliations:** Mental Health Program, Eastern Health, Box Hill, VIC, Australia; Eastern Health Clinical School, 2541 Monash University, Box Hill, VIC, Australia; Centre of Mental Health Education and Research, 95903 Delmont Private Hospital, Burwood, VIC, Australia; Department of Psychiatry, 2541 Monash University, Clayton, VIC, Australia; Department of Psychiatry, 2281 The University of Melbourne, Melbourne, VIC, Australia; Department of Psychiatry, 2541 Monash University, Clayton, VIC, Australia; Mental Health Program Monash Health, Clayton, VIC, Australia; Centre of Women’s and Children’s Mental Health, Melbourne, VIC, Australia; Department of Psychiatry, University of Melbourne, VIC, Australia; Peninsula Health, Frankston, VIC, Australia; 2541 Monash University, Clayton, VIC, Australia; 2541 Monash Medical Centre, Clayton, VIC, Australia; Centre of Mental Health Education and Research, 95903 Delmont Private Hospital, Burwood, VIC, Australia

**Keywords:** antidepressants, intranasal esketamine, N-methyl-D-aspartate antagonist, treatment resistant depression

## Abstract

**Objective:**

To review the usefulness of esketamine for treatment-resistant depression.

**Method:**

Pivotal trials of intranasal esketamine in treatment-resistant depression were synthesized as a narrative review.

**Results:**

Esketamine is postulated to act through antagonism of N-methyl-D-aspartate (NMDA) glutamate receptors, but opioidergic effects may also be involved. Unlike intravenous ketamine, esketamine is given intranasally (under clinical observation), usually in addition to an oral antidepressant. Trials compared esketamine plus antidepressant versus placebo plus antidepressant. At 4 weeks, remission was 37% higher with esketamine/antidepressant than placebo/antidepressant. Speed of response and improvement in suicidality were comparable. In stable remitters on esketamine/antidepressant, 45% relapsed when esketamine was withdrawn over the following 6 months (whereas 25% relapsed on esketamine/antidepressant). Response appears less likely in patients with multiple antidepressant failures. Adverse effects include dissociation, dizziness, nausea, sedation, and headache but no psychosis. Hypertension affected 13%, especially older patients. Dose frequency is twice-weekly for 4 weeks, then weekly/fortnightly thereafter. No abuse has been reported. Unsubsidised cost may be beyond the reach of many Australians.

**Conclusion:**

Intranasal esketamine plus antidepressant has been approved by regulators as moderately effective and acceptably tolerable for treatment-resistant depression. Cost is a drawback. Use often needs to be long-term and vigilance for abuse is essential.

Treatment-resistant depression (TRD), usually defined as inadequate response to two or more antidepressants, is associated with serious morbidity and mortality including suicide.^1^ Ketamine has emerged as a potential new treatment for TRD.^2^ An arylcyclohexylamine derivative of the hallucinogen phencyclidine, ketamine was introduced as a dissociative anaesthetic in the 1960s. From 2000, subanaesthetic doses of intravenous ketamine were observed to induce antidepressant effects within 4 h, but persistence of effects required repeated infusions and many patients experienced dissociation and psychotic symptoms.^2^

Among a broad spectrum of neurochemical effects, ketamine modulates activity of the excitatory amino acid glutamate through non-competitive antagonism at the NMDA receptor on gamma-amino-butyric-acid inter-neurones. Glutamate requires the presence of either glycine or D-serine to be effective. This activates α-amino-3-hydroxy-5-methyl-4-isoxazolepropionic acid (AMPA) receptors.^2^ Subsequent intracellular effects involve gene expression and production of molecules such as brain-derived neurotrophic factor (BDNF).^2^

While ketamine appears to share the downstream antidepressant mechanism of action with other antidepressants through changes in brain structure, the initial rapid antidepressant effect may be due to its complex neuromodulatory effects through the NMDA receptor, or possibly through effects on opioid receptors.^2^ Naltrexone (a mu-opioid receptor antagonist) blocks the antidepressant but not dissociative effects of ketamine.^3^ That ketamine may exert an antidepressant effect through an opioidergic mechanism is relevant for concern about its abuse potential, but recent findings suggest that the neuromodulatory effects of ketamine require an interactive effect between NMDA and opioid mechanisms.^4^

Ketamine is a racemic mixture of equal quantities of two enantiomers: S (sinister)-ketamine and R (rectus)-ketamine. Racemic ketamine is available, but not approved for use in depression in by the Therapeutic Goods Administration (TGA). In March 2021, the TGA approved the use of intranasal esketamine (in conjunction with a newly initiated antidepressant) in adult patients with major depression who have not responded adequately to at least two different antidepressants in adequate dose and of adequate duration to treat the current moderate to severe depressive episode.^5^ Given that one in three patients do not respond to available oral treatment^6^ and two thirds of patients treated with antidepressants do not fully recover,^7^ esketamine is of interest to both patients and clinicians. This paper briefly reviews and discusses the usefulness of esketamine in clinical management of unipolar treatment resistant depression.

## Method

A search of randomized controlled trials (RCTs) of intranasal/nasal spray esketamine in the treatment of refractory/resistant depression was conducted using PubMed, to identify pivotal phase 3 publications. Post-hoc studies were excluded. The results were synthesized as a narrative review.^8^

## Results

### Efficacy evidence

The three pivotal acute studies with intranasal esketamine^9–11^ were designed as “add-on” studies, as required by the US Food and Drug Administration. Participants had failed at least two previous antidepressants; two studies examined adults; one included persons over 65 years. All participants were commenced on a new antidepressant, either a selective serotonin reuptake inhibitor (SSRI) or serotonin noradrenaline reuptake inhibitor (SNRI) and randomised to either intranasal esketamine or placebo. All comparisons were between intranasal esketamine plus SSRI/SNRI or placebo plus SSRI/SNRI, with flexibility in dose. A limitation of the studies was the assumption of equivalence between the new antidepressants commenced.

Intranasal esketamine doses were 54 or 86 mg twice weekly for 28 days. Over 85% of patients completed the studies, and overall improvements in depression were moderate in both esketamine/antidepressant and placebo/antidepressant groups. Importantly, two of the studies failed to differentiate in efficacy between esketamine and placebo,^10,11^ while the third study demonstrated esketamine was more effective than placebo but to a clinically small degree (MADRS 95% CI −7.31 to −0.64).^9^ Esketamine did not demonstrate a difference from placebo in reduction of depression at 24 h, or at 2 days. Suicidal thinking improved considerably with both treatment conditions, but similarly there were no differences between esketamine and placebo in the first 24 h or at any other time during the study.^2,6^

In a further 12-month study, responders or remitters to esketamine/antidepressant in the short term were either blindly continued on treatment or changed to placebo/antidepressant. Continuing on treatment decreased the risk of relapse by 51% in remitters and 70% in responders compared to placebo/antidepressant; however, 25% of patients taking esketamine/antidepressant suffered relapse of depression despite continuing treatment.^12^

Following the equivocal pivotal studies, a systematic review of TRD added a further four RCT studies and a maintenance safety study (Table 1). The extra studies used a comparable methodology, and two studies examined patients with suicidal ideation. A meta-analysis of the pooled results of the seven efficacy trials (*n* = 1421) showed that at four weeks patients on esketamine/antidepressant had a 22% higher chance response (>50% improvement) and 37% higher chance of remission than those on placebo/antidepressant.^7^ A further review showed the number needed to treat (NNT) was six for response and seven for remission, which is consistent with moderate efficacy.^2^ Efficacy of intranasal esketamine was not established in patients over aged 65. Esketamine was more effective in patients with fewer previous antidepressant failures.^2^

**Table 1.** Summary of 28 days efficacy of phase 3 esketamine studies (adapted from Jawad et al. 2021^7^)

Author/year	Study n	Dose	Population	Response at week 4	Remission (MADRS <12) Esk vs pbo
Canuso C et al., 2018^14^	68	84 mg	19-64 years, MDD & SI	NS	60% vs 42%
*Popova V et al., 2019^9^	223	56, 84 mg	18-64 years TRD	MADRS group difference −4.0 (CI −7.31 to −0.64)	53% vs 31%
*Fedgchin M et al., 2019^10^	346	56, 84 mg	18-64 years TRD	MADRS group difference −4.0, NS	38% vs 31%
*Ochs-Ross R et al., 2020^11^	137	28, 56, 84 mg	>65 years TRD	MADRS group difference −3.6 NS	18% vs 7%
Fu D et al., 2020^15^	224	56, 84 mg	18-64 years MDD with SI	NS	54% vs 38%
Takahasi N et al., 2021^16^	202	28, 56, 84 mg	20-64 years TRD	NS	19% vs 21%
Ionescu D et al., 2021^17^	227	84 mg	18-64 years MDD with SI	NS	47% vs 37%

^*^Pivotal 4 weeks study.

MADRS: Montgomery–Asberg depression rating scale; MDD: major depressive disorder; SI: suicidal ideation; NS: not significant; Esk: eskatamine; Pbo: placebo.

### Safety evidence

In the five pivotal studies (including two long-term^12,13^) of intranasal esketamine plus antidepressant, the most common adverse effects were dissociation, dizziness, nausea, sedation and headache (Table 2). Most reactions were mild to moderate and resolved on the day of administration. Blood pressure elevations were mostly minor and resolved within 2 hours of administration. However, 17% of patients over 65 experienced a transient systolic blood pressure increase of ≥40 mmHg.^5^

**Table 2.** Rates of common side effects of esketamine compared to placebo^5^

	Esketamine & SSRI/SNRI (%)	Placebo & SSRI/SNRI (%)
Dissociation	40	6
Dizziness	37	7
Nausea	27	6
Sedation	25	7
Headache	24	12
Vertigo	18	3
Dysgeusia	17	11
Hypoaesthesia	17	1
Blood pressure increase	13	4
Anxiety	13	6
Vomiting	10	1

SSRI: selective serotonin reuptake inhibitor; SNRI: serotonin noradrenaline reuptake inhibitor.

Sedation usually resolved within 1.5 h of administration and cognitive impairment within 2 h. Two patients in the pivotal studies became unconscious (one repeatedly, the other unpredictably), supporting the need for monitoring patients post administration until clinically stable.^18^ In use up to 12 months, it was observed that tolerance for dissociation gradually develops, and there was no indication of lasting neurocognitive impairment (which occurs with ketamine abuse). No hepatic dysfunction or ulcerative cystitis was observed.^13^ Substance abuse was not observed to develop during studies with esketamine. Post marketing surveillance and analysis of web-based forum content has identified some indications of craving, tolerance, withdrawal, and co-use of illicit psychedelics.^19^ Women report more adverse effects than men (although sex differences in efficacy were not observed).^20^

### Using esketamine

Esketamine is rapidly absorbed intranasally and peak concentrations are reached after 20 to 40 min. Metabolism is hepatic, primarily via CYP2B6 and CYP3A4. Half-life is 7 to 12 h^5.^ After administration, blood pressure needs to be monitored for at least 40 min or until in the normal range and general observations should continue until the patient is stable (adverse effects generally disappear within 2 hours). Patients should not eat for 2 hours or drink for 30 min prior to administration, due to possible nausea and vomiting.^5^

Esketamine is only available through approved centres. There are three doses: 28 mg, 56 mg and 84 mg, although the evidence for the efficacy of the 28 mg dose is limited. In the long-term most patients will require 84 mg. Each treatment costs between $400 and $1000 depending on dose, plus administration costs and there is no government subsidy. Frequency starts at twice weekly for 4 weeks, and eventually becomes weekly or every 2 weeks, and may need to be indefinite.

## Discussion

Although the short term pivotal esketamine studies struggled to show a difference between esketamine and placebo due to the regulatory antidepressant commencement requirement, longer term evidence and meta-analytic data have persuaded the TGA, as well as regulators in the United States, United Kingdom and Europe, of the drug’s efficacy as an antidepressant adjunct in treatment resistant depression.^2^ The NNT of six for response and seven for remission is similar to that for antidepressant drugs.

Tolerability is also acceptable though not without challenges such as transient hypertension, particularly in older patients. Unlike ketamine, intranasal esketamine does not cause psychotic symptoms. However, the tendency for dissociation requires patients to be carefully prepared for the experience, and supported during esketamine administration, in order to avoid severe anxiety and distressed behaviour.

Clinically, the comparative benefit of intranasal esketamine is not clear. Although esketamine plus antidepressant was observed to improve symptoms of depression and reduce suicidal thinking within the first 24 h after administration, no advantage over placebo plus antidepressant could be demonstrated. Additionally, whether esketamine added to antidepressant therapy in treatment resistant depression is superior to established antidepressant adjunctive treatments (such as aripiprazole and quetiapine) will require appropriate head-to-head double-blind investigations.^18^

Of concern is the absence of longer-term experience with esketamine, particularly with respect to the possibility of cumulative neurocognitive dysfunction.^2^ In view of the regulated method of esketamine administration, it is unlikely that esketamine itself will be abused. However, possible promotion of illicit substance abuse has been raised in post marketing surveillance,^19^ and may be reinforced by the anecdotally low price of street ketamine.^21^

The cost of esketamine and its administration is substantial. Administration in approved centres, the need for a period of observation and monitoring adds to the cost. Other practical barriers include driving restrictions on the day, distance from a treatment centre, cost of patient transport and low availability outside working hours.

## Conclusion

Intranasal esketamine is modestly effective in treatment resistant depression when added to an antidepressant and has reasonable tolerability. The logistics of administration and subsequent observation will be onerous for some patients. The cost of esketamine and its administration is substantial. There are concerns about abuse potential as a consequence of esketamine treatment. Given these reservations, the current clinical utility of intranasal esketamine remains limited.
